# t(8;14;18): A 3-way chromosome translocation in two patients with Burkitt's lymphoma/leukemia

**DOI:** 10.1186/1476-4598-6-35

**Published:** 2007-06-04

**Authors:** Delong Liu, Josif Shimonov, Suneeta Primanneni, Yongrong Lai, Tauseef Ahmed, Karen Seiter

**Affiliations:** 1 Division of Oncology/Hematology, New York Medical College and Westchester Medical Center, Valhalla, NY 10595, USA; 2 Department of Medicine, Richmond University Medical Center, Staten Island, NY 10310, USA; 3 Department of Hematology, First Affiliated University Hospital, Guangxi Medical University, Nanning, Guangxi Province, China

## Abstract

Burkitt's lymphoma (BL) is a heterogeneous group of highly aggressive mature B-cell malignancies. It is characterized by a high rate of turnover of malignant cells and deregulation of the c-myc gene. It is typically associated with a t(8;14) translocation. Dual translocation of t(8;14) (c-myc) and t(14;18) (bcl-2) was reported to be associated with extremely poor prognosis. This study reports a novel t(8;14;18) triple translocation in two patients with Burkitt's lymphoma.

## Background

Burkitt's lymphoma/leukemia (BL) is a heterogeneous group of highly aggressive mature B-cell malignancies. It is characterized by a high rate of turnover of malignant cells and deregulation of the c-myc gene. The BL tumor cells usually express the B-cell-specific surface markers CD19, CD20, together with surface immunoglobulin (Ig), as well as CD10. The histologic hallmark of BL is the presence of apoptotic cells within scattered macrophages, a feature responsible for the "starry sky" microscopic appearance. A characteristic chromosome translocation associated with this disease typically takes place between chromosome 8q24 (c-myc) and one of the Ig gene-containing chromosomes, 14q32 (Ig H gene), 2p12 (Ig *kappa*) or 22q11 (Ig *lambda*). The frequency of such translocation is estimated to be approximately 80% t(8;14), 15% t(2;8), and 5% t(8;22), respectively [1]. We report in this study two cases of BL with a novel three-way chromosome translocation, t(8;14;18).

## Results and discussion

### Case 1

**Figure 1:**
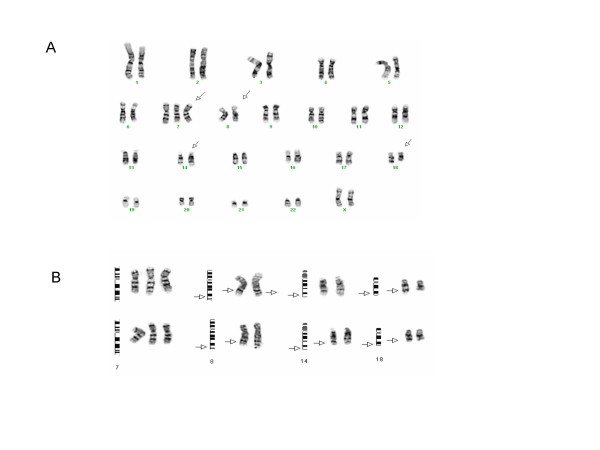
Cytogenetic abnormalities in case 1. (A). Karyotype 47, XX, +7, t(8;14;18) (q24.1;q32;q22). Arrows indicate abnormal chromosomes. (B). Diagramatic representation of +7 and 3-way chromosome translocations. Arrows indicated abnormal chromosome regions involved in the 3-way translocation.

A 61 year- old Arabic female presented with abdominal pain, weakness and fever in April, 1997. Physical examination was significant for splenomegaly. Her laboratory revealed WBC 2.7 × 10^9^/L, Hgb 107 g/L, platelets 178 × 10^9^/L, and LDH 1250 u/L. CT scan confirmed splenomegaly. Bone marrow was hypercellular with 30% lymphoid blasts. Flowcytometric analysis revealed a B cell population expressing CD19, CD20, CD22, HLA-DR and *kappa*, but negative for CD5, CD10, CD23, and CD34, and tdT. The chromosome analysis of the bone marrow revealed 47, XX, +7, t(8;14;18) (q24;q32;q22) (Fig. 1). This was consistent with BL by WHO criteria. She was given chemotherapy with cyclophosphamide, doxorubicin, vincristine, and prednisone following the L-20 regimen [2,3]. The patient was in complete remission (CR) and received maintenance chemotherapy. In November, 2002 she felt abdominal pain without any other complaint. Physical examination was unremarkable. WBC 4.7 × 10^9^/L, Hgb 115 g/L, and platelets 215 × 10^9^/L. CT scan revealed marked intra-abdominal lymphadenopathy. Bone marrow smear and flowcytometric analysis did not show overt involvement by BL, though focal involvement of the bone marrow was demonstrated on immunohistochemical study. The cytogenetic analysis from the bone marrow sample revealed only 46 XX. She was given the intensive chemotherapy with rituxamab plus hyper-CVAD [4]. She also received radiation therapy to the residual mass. She subsequently completed two years of maintenance therapy in August 2005 and has remained in CR at the time of this report.

### Case 2

**Figure 2:**
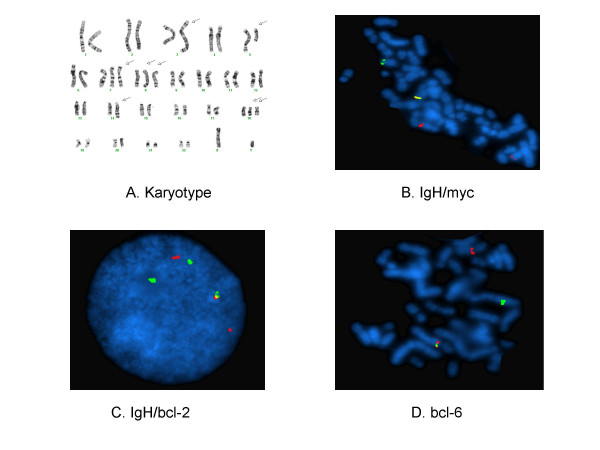
Cytogenetic abnormalities in case 2. (A). Karyotype 44–49, XY, t(3;5) (q27;q15), t(8;14;18) (q24;q32;q21), +der (8) t(8;14;18), +der(18) t(8;14;18). Arrows indicate abnormal chromosomes. (B) IgH/myc gene fusion. (C). IgH/bcl-2 gene fusion. (D). bcl-6 gene rearrangement.

A 56 year- old Hispanic male went to ER with worsening left sided facial pain with erythema and edema. Physical examination was significant for lymphadenopathy in the neck and inguinal areas as well as splenomegaly. He also had decreased air entry in both lungs and bilateral ankle edema. His CBC revealed WBC 28.4 × 10^9^/L, Hgb 109 g/L, and platelets 330 × 10^9^/L. The chemistry panel was significant for BUN 58 mg/dL, Creatinine 4.7 mg/dL, LDH > 2500 u/L, Uric acid 27.8 mg/dL. CT scan revealed bilateral pleural effusions, splenomegaly, massive matted retroperitoneal lymphadenopathy and bilateral inguinal lymphadenopathy. MRI of the brain showed a 5 cm left pterygoid muscle mass extending into the cavernous sinus and lateral aspect of the orbit. Bone marrow was hypercellular and partially replaced by intermediate to large-sized neoplastic lymphoid cells with cytoplasmic vacuoles. Flowcytometric analysis revealed a B cell population expressing CD19, CD20, CD22, CD10(dim), surface IgG, HLA-DR and CD43, but negative for CD5, CD34, and tdT. The chromosome analysis of the bone marrow revealed 44–49, XY, t(3;5) (q27;q15), t(8;14;18) (q24;q32;q21), +der (8) t(8;14;18), +der(18) t(8;14;18) (Fig. 2). FISH study was positive for gene rearrangement of bcl-6, and fusions of myc, IgH, and bcl-2 (Fig. 2). This was consistent with BL. He received hemodialysis for his acute renal failure. He was given the intensive chemotherapy with rituximab plus hyper- CVAD. His lymphadenopathy resolved on examination. His renal function returned to normal quickly, and his blood counts returned to normal range upon discharge from the hospital.

This study identified two cases of BL with this novel t(8;14;18) triple translocation from one institution over 10 years. The first patient relapsed five and a half years after initial diagnosis, but her disease remained to be chemo-sensitive. Gene locus translocations of myc, IgH, and bcl-2 were confirmed by FISH analysis in the second case. After extensive literature search and review, we believe this is the first report of t(8;14;18) triple translocation associated with clinical cases (Table 1). A review of a large cytogenetic database from 1350 leukemia and lymphoma karyotypes disclosed eight cases with karyotypes of t(8;14) and t(14;18) involving 3-way recombinations of MYC-IGH-BCL2 [5]. Interestingly, 3-way recombinations of MYC-IGH-BCL2 were demonstrated by multicolor FISH and locus-specific FISH analysis [5]. It is therefore possible that the 3-way recombinations of MYC-IGH-BCL2 may take place more often than it is reported, since the multicolor FISH and locus-specific FISH are not routinely done. It appears nonetheless that this 3-way chromosome translocation is an extremely rare genetic event occurring in clinical cases. There have been three separate reports of t(8;14;18) 3-way translocation that were found in BL cell lines [6-8]. Dual translocations of t(8;14) and t(14;18) involving c-myc and bcl-2 have been reported in Burkitt-like lymphoma patients [9-12]. 13 such patients with the dual translocation were found to have rapid clinical course and extremely poor prognosis [9]. None of the 13 patients survived past 7 months. Three cases of non-Burkitt lymphoma in the same report had triple translocations of t(1;8;22), t(1;14;18), and t(12;14;18), respectively [9]. t(7;8;14) was found in BL cell line, CA46 [8]. In another cell line of BL, a novel gene, bcl7a, from the triple translocation t(8;14;12) was identified [13]. c-myc translocation can also be seen in diffuse large B-cell lymphoma (DLBCL). DLBCL with c-myc translocation can be difficult to reliably differentiate from BL by currently available diagnostic tools [14]. Through gene expression profiling, eight cases of pathologically-diagnosed DLBCL and one case of high-grade lymphoma were reclassified as BL. These nine cases all expressed c-myc, bcl-2, and had high Ki-67 scores [14]. It is crucial to distinguish between DLBCL and BL, since the overall survival of those BL patients who were treated with intensive regimens were superior to those who received CHOP-like regimens [14]. Regardless of pathological diagnosis of BL or Burkitt-like lymphoma, it seems that poor-prognostic patients with dual and triple translocations involving c-myc and bcl-2 should receive intensive chemotherapy [2,3]. It is unclear whether hematopoietic stem cell transplantation plays any role in the therapy of BL with complex chromosome translocations [15-18].

**Table 1:** 3-way translocations associated with Burkitt's lymphoma/leukemia

Author	Karyotype	*Genes	Source	Reference
Ooteghem, 1994	t(8;14;18)	bcl-2	cell line	6
Dyer,1996	t(8;14;18)	NR	cell line	7
Zimonjic, 2001	t(8;14;18)	NR	cell line	8
	t(7;8;14)	NR	cell line	8
Zani,1996	t(8;14;12)	bcl-7a	cell line	13
Macpherson,	t(1;8;22)	NR	patient	9
1999	t(1;14;18)	NR	patient	9
	t(12;14;18)	NR	patient	9
Liu, 2007	t(8;14;18)	NR	patient	TR
	t(8;14;18)	bcl-2	patient	TR

*genes: gene mutation in addition to c-*myc*. NR: not reported. TR: this report

## Abbreviations

BL: Burkitt's lymphoma/leukemia; FISH: fluorescent in situ hybridization; CHOP: cyclophosphamide, adriamycin, vincristine, prednisone.

## Competing interests

The author(s) declare that they have no competing interests.

## Authors' contributions

DL, KS conceived the study. DL, JS and SP collected data. DL, JS, SP, YL, TA and KS coordinated the design. DL drafted the final manuscript.
